# A scavenger receptor B (CD36)-like protein is a potential mediator of intestinal heme absorption in the hematophagous ectoparasite *Lepeophtheirus salmonis*

**DOI:** 10.1038/s41598-019-40590-x

**Published:** 2019-03-12

**Authors:** Erna Irene Heggland, Christiane Eichner, Svein Isungset Støve, Aurora Martinez, Frank Nilsen, Michael Dondrup

**Affiliations:** 1 Department of Biological Sciences & Sea Lice Research Centre (SLRC), University of Bergen, Bergen, Norway; 2 Department of Biomedicine & K.G. Jebsen Centre for Neuropsychiatric Disorders, University of Bergen, Bergen, Norway; 3 Department of Informatics & Sea Lice Research Centre (SLRC), University of Bergen, Bergen, Norway

## Abstract

Intestinal absorption of heme has remained enigmatic for years, even though heme provides the most bioavailable form of iron. The salmon louse, *Lepeophtheirus salmonis*, is a heme auxotrophic ectoparasite feeding on large quantities of blood from its host, the salmon. Here we show that a scavenging CD36-like receptor is a potential mediator of heme absorption in the intestine of the salmon louse. The receptor was characterized by a heme binding assay using recombinantly expressed protein, *in situ* hybridization and immunohistochemistry, as well as functional knockdown studies in the louse. A computational structural model of the receptor predicted a binding pocket for heme, as also supported by *in silico* docking. The mRNA and protein were expressed exclusively in the intestine of the louse. Further, knocking down the transcript resulted in lower heme levels in the adult female louse, production of shorter egg strings, and an overall lower hatching success of the eggs. Finally, starving the lice caused the transcript expression of the receptor to decrease. To our knowledge, this is the first time a CD36-like protein has been suggested to be an intestinal heme receptor.

## Introduction

Iron is a transition metal, which is essential for many proteins present in all branches of the phylogenetic tree of life and must be obtained through the diet. The most bioavailable form of iron is heme, the iron-containing pyrrole ring of protoporphyrin IX^1^. Heme is a prosthetic group found as cofactor in many metalloproteins and is known to contribute to essential cellular processes, such as electron transport, signal transduction, detoxification, gas transport and sensing^2–4^. Although heme is necessary for many purposes in the cell, it may also exert cytotoxic effects by generation of reactive oxygen species (ROS) and cause damage to DNA, proteins and lipids^5–7^.

The classical heme biosynthetic pathway is an evolutionarily conserved multi-step enzymatic reaction that in eukaryotic cells takes place partially in the mitochondria and partially in the cytoplasm. Heme biosynthesis begins with the synthesis of δ-aminolevulinic acid (ALA) by δ-aminolevulinate synthase 1 (ALAS1) as the rate-limiting reaction, and ends with the addition of an iron atom to the center of the protoporphyrin IX ring by ferrochelatase (FECH)^8^. Even though heme is essential for aerobe cells, some organisms are unable to produce this cofactor on their own. Natural heme auxotrophic organisms depend upon exogenous heme through their diet for survival. In this group we find, among others, the hematophagous parasitic cattle tick *Rhipicephalus (Boophilus) microplus* feeding off cattle blood^9^, the soil-nematode, *Caenorhabditis elegans*, and the parasitic nematode *Brugia malayi*^10^.

By analyzing its genomic sequence, we uncovered that the Atlantic salmon louse, *Lepeophtheirus salmonis*, is likely among the natural heme auxotrophs as well, because it lacks homologs for most enzymes of the classical heme biosynthetic pathway, including ALAS1 and FECH (https://licebase.org, unpublished). The salmon louse is an obligate ectoparasite of salmonid fish. It is considered a major problem for both farmed and wild salmon populations, and as resistances to various treatments have occurred recently, effective methods for pest control are in high demand^11^. The life cycle of this parasite is divided into eight stages separated by molts. These are in chronological order: nauplius I and II, copepodid, chalimus I and II, preadult I and II, and adult lice^12,13^. The developmental pace of *L. salmonis* correlates for the most part with temperature. At 10 °C, development from fertilization to mature adult lice is completed in approximately 40 (♂) to 52 (♀) days^14^. From hatching and until it reaches the infectious copepodid stage, *L. salmonis* is planktonic and survives on energy reserves from the yolk sac. When these eventually wear down, the copepodid has to infect a salmonid host in order to complete its life cycle. Once attached to a suitable host, *L. salmonis* feeds off the host’s skin and blood^15^. By hematophagy, the parasite is exposed to significant amounts of hemoproteins and other nutrients.

The salmon louse is likely dependent on its vertebrate host for heme supply; consequentially there needs to exist a way of absorbing heme from ingested blood within the digestive tract of the parasite. However, heme transport through the cell membrane as well as intra- and intercellular heme trafficking are generally poorly understood. An organism lacking endogenous heme provides the opportunity to study trafficking of the cofactor without further confounding effects by endogenous cellular synthesis. In the heme auxotroph *C. elegans*, heme transporter HRG-1 transmembrane proteins mediate heme uptake and homeostasis. Whereas HRG-1 primarily localizes to endosomal and lysosomal organelles, its paralogue HRG-4 localizes to the plasma membrane and is expressed in the intestine; therefore HRG-1 and HRG-4 might act in a concerted fashion to take up environmental heme^16^. However, the *hrg-4* gene is nematode-specific implying that different mechanisms of heme uptake exist in other animal species. The mammalian proton-coupled folate transporter/heme carrier protein 1 (PCFT/HCP1) was also initially proposed as an intestinal heme transporter^17^, but its function has since been debated, and later research characterized PCFT/HCP1 as a folate transporter with at best minor affinity to heme^18^.

Limited success in identifying an evident candidate for the function of intestinal heme absorption has led to a shift in the traditional thinking as to which characteristics a heme receptor or transporter should fulfill. As the porphyrine ring is an amphipathic molecule with both polar and nonpolar properties, it has been suggested that it may be trafficked, alike lipids, via membrane-tethered complexes, lipid transfer proteins, vesicular trafficking, or lipid transporters^19^. A gene found to be highly expressed in the salmon louse intestine encodes a CD36-like protein. By sequence similarity, it belongs to the scavenger receptor class B (SCARB) family. Proteins of the SCARB family consist of two transmembrane domains, an extracellular ligand-binding domain, and short intracellular N- and C-terminal tails. Mammalian homologous proteins have been reported to scavenge a large variety of ligands (albeit not including heme), e.g. various lipoproteins such as oxidized and non-oxidized LDL^20^, beta-carotene^21^, and very long chain fatty acids^22^.

In this study, we characterize the SCARB-like (*LsHSCARB*) gene and protein found in *L. salmonis* and hypothesize that LsHSCARB facilitates heme uptake across the intestinal membrane. Localization of the gene and protein, structural analysis, functional knockdown studies and a recombinant binding assay support our hypothesis. Our data support the existence of a novel pathway of heme scavenging from the arthropod intestine, and yield a potential new drug target for sea lice control.

## Results

### Sequence analysis

**Figure 1:**
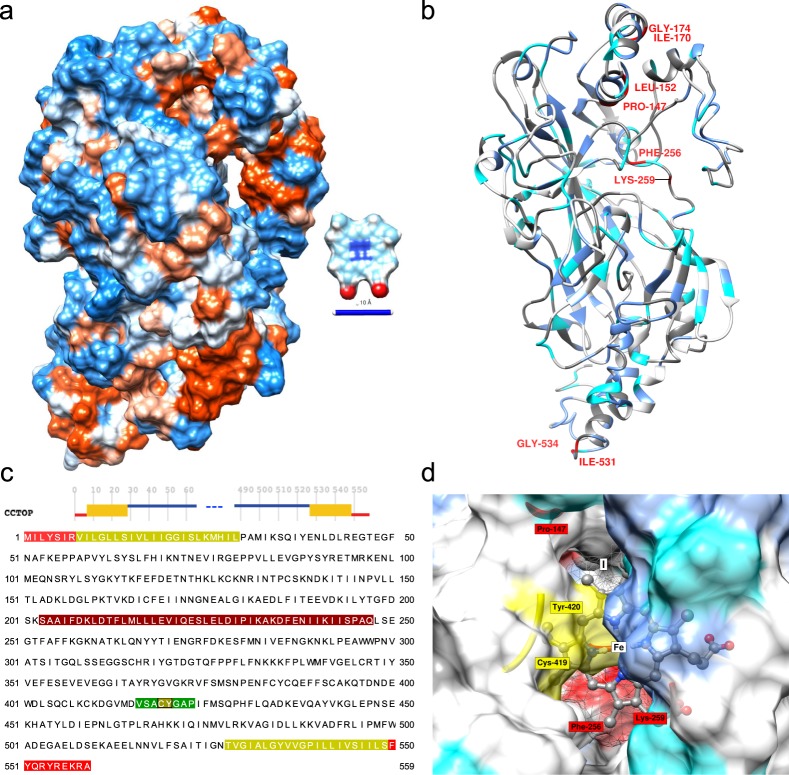
Structural *de novo* prediction for the protein sequence of LsHSCARB. Best scoring tertiary structure model generated from i-TASSER depicted as (**a**) hydrophobic surface (red: hydrophobic, blue: hydrophilic) The ligand protoporphyrin IX (C34H32N4O4, ZINC26671872) is depicted for comparison, scale bar = 10 Å. (**b**) ribbon representation with predicted secondary structure, and annotated residues with potential implication in heme binding (dark gray: aromatic, blue: aliphatic, cyan: potential axial ligand (C, M, Y, K, or H), red: residues predicted by HemeBIND). (**c**) Consensus membrane topology prediction by CCTOP over residues and sequence, red: intracellular, orange-green: trans-membrane, blue: extracellular domain, dark-red: lateral hydrophobic surface of the hydrophobic pocket, green: interior surface of predicted binding pocket. (**d**) Results from Receptor-Ligand docking by AutoDock Vina with the best-scoring (I) docking position. The highest scoring viable docking position is located inside the hydrophobic pocket, Cys-419 and Tyr-420 (yellow) are proximal to the hypothetical iron center of a heme ligand (Fe), residues are in same color code as in (b).

The full salmon louse heme scavenger (*LsHSCARB*) cDNA sequence (Ensembl metazoa stable ID: EMLSAG00000005382, GenBank accession: CDW29028.1) was verified by 5′ and 3′ Rapid amplification of cDNA ends (RACE) PCR using sequence specific primers (Table 1). The gene consists of a 220 bp 5′-untranslated region (UTR), an ORF of 1677 bp and a 288 bp 3′-UTR. The ORF translates into a 559 amino acid (aa) long protein with two predicted transmembrane domains in the N- and C-terminal (from aa 7 → 28 and from aa 528 → 549) with the terminals located intracellularly (Fig. 1c), and a signal anchor sequence from aa 1 → 23. The protein has a predicted N-linked glycosylation site at aa 260 and three predicted O-linked glycosylation sites at aa 302, 303 and 376. The sequence of the ORF was analyzed in InterproScan, and was characterized as belonging to the cluster of differentiation 36 (CD36) family (PF01130). A protein BLAST search of the *L. salmonis* full length protein showed 31% identity with a scavenger receptor class B1 in the kuruma prawn, *Marsupenaeus japonicus* (GenBank accession: AKO62849), and 29% identity with the freshwater shrimp, *Macrobrachium nipponense* scavenger receptor B1 (GenBank accession: ALK82306).

**Table 1:** Primers used for RACE, *in situ* hybridization (ISH), RNA interference (RNAi) and qPCR.

Primer name	Sequence (5′→3′)	Application
*LsHSCARB* 5′ RACE	CCTCCTTCCACTTCCACTTCGGACTCA	5′ RACE
*LsHSCARB* 3′ RACE	GTCAAGAATTTTTCTCATGCGCCAA	3′ RACE
*LsHSCARB* fwd	AGCGGATAAACTCGATGGCT	ISH/RNAi
*LsHSCARB* T7 fwd	TAATACGACTCACTATAGGGAGAAGCGGATAAACTCGATGGCT	ISH/RNAi
*LsHSCARB* rev	TTTGCTTGGCGCATGAGAAA	ISH/RNAi
*LsHSCARB* T7 rev	TAATACGACTCACTATAGGGAGATTTGCTTGGCGCATGAGAAA	ISH/RNAi
Cod_CPY185 fwd	TAATACGACTCACTATAGGGATAGGGCGAATTGGGTACCG	RNAi
Cod_CPY185 rev	TAATACGACTCACTATAGGGAAAGGGAACAAAAGCTGGAGC	RNAi
SYBR *LsEF1α* fwd	GGTCGACAGACGTACTGGTAAATCC	qPCR
SYBR *LsEF1α* rev	TGCGGCCTTGGTGGTGGTTC	qPCR
SYBR *LsHSCARB* fwd	TCCGCTTGATCCCCATGTTC	qPCR
SYBR *LsHSCARB* rev	GCCAACGACATAGCCAAGAGC	qPCR

T7 promoter extension is underlined.

### Homology modelling and heme binding

A three-dimensional model of LsHSCARB was predicted using i-TASSER (Fig. 1a,b). The highest scoring model I (S-score = −1.33) presents with a lateral cavity in the extra-cytoplasmic domain that is enriched with hydrophobic residues. By sequence alignment with the highest ranked threading template (4F7B, Lysosomal Integral Membrane Protein, LIMP-2, RMSD = 0.56, Supplementary Fig. S1) the presence of a hydrophobic loop (residues 202–236: SAAIFDKLDTFLMLLLEVIQESLELDIPIKAKDF, Fig. 1a–c) is detected within an insertion in LsHSCARB (Supplementary Fig. S2). The opposite surface of the cavity consists of a loop region (residues 416–423: VSACYGAP, Fig. 1c) with central Cys-419 Tyr-420 (CY) residues (Fig. 1d).

Using HemeBIND as a specialized predictor of heme-binding residues to integrate sequence and structural information following residues were predicted as potential heme interacting: Pro-147, Leu-152, Ile-170, Gly-174, Phe-256, Lys-259, Ile-531, and Gly-534 (Fig. 1b). While Ile-531 and Gly-534 are located in the intracellular part of the receptor, all other residues belong to the predicted extracellular domain.

We then performed docking simulations of the generated model I using protoporphyrin IX (PPOIX) as the ligand in AutoDock Vina. The best viable docking pose is predicted inside the hydrophobic pocket with the CY residues extending over the iron-center of heme (Vina score: −9, Fig. 1d, Supplementary Data S1–4). To assess whether the observed scores are comparable to docking results with known hemoproteins and other heme-binding proteins, we analyzed the best docking poses of ten experimentally obtained structures with heme ligand after removing the heme moiety and re-docking with PPOIX (Supplementary Table S1). Nine out of ten docking attempts placed the ligand at a distance of <2 Å between centroids, and six out of ten attempts resulted in an RMSD <1 Å between the docking pose and the experimental structure. The only failed docking attempt (3GNF, Cytochrome c-553 from *Bacillus pasteurii*) also presented with the worst Vina score of all experiments. All successful re-docking attempts to heme-proteins presented with a Vina score ≤−9. In addition, we tried docking PPOIX with two alternative PDB structures of LIMP-2, one of which was used as a threading template by i-TASSER to generate the LsHSCARB homology model. To the best of our knowledge, LIMP-2 has not been previously reported as a heme-interacting protein. These resulted in very similar optimal VINA scores of −7.6 and −7.7 respectively (Supplementary Table S1).

When comparing *in silico* docking results and HemeBIND predictions, three out of six predicted extracellular residues are within a distance of <2 Å from the electrostatic surface of the docked ligand, Lys-259 interacts directly with the ligand surface, whereas Pro-147 and Phe-256 are proximal to the docking site (Fig. 1d)

**Figure 2:**
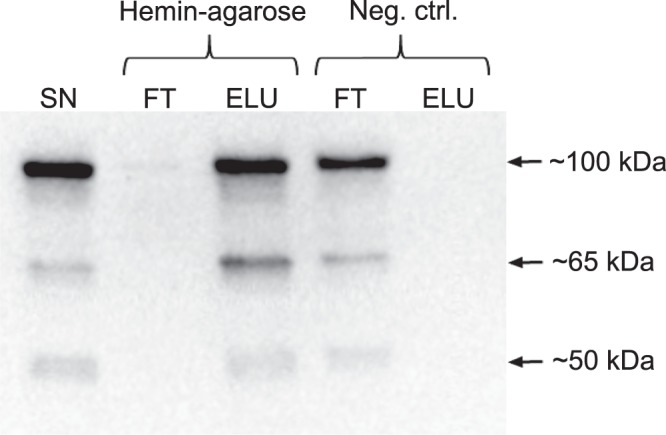
Hemin-agarose pull-down assay. Cell lysates of *E. coli* Bl21 cells expressing LsHSCARB (residues 31–523) were mixed with hemin-agarose (Sigma-Aldrich), and samples analysed by western blot using an anti-LsHSCARB antibody (Genscript). LsHSCARB was detected in the supernatant (SN), the elution (ELU) of hemin-agarose, and the flow through (FT) of the negative control. Exposure time = 29.5 sec. The experiment was conducted three independent times with similar results.

In order to investigate whether LsHSCARB was able to bind heme directly, we expressed the extracellular part of LsHSCARB (residues 31–523) in *E. coli* Bl21 De3 cells and investigated its ability to bind hemin-conjugated agarose resin. Cells were lysed and the supernatant containing the recombinant protein was incubated with either hemin-conjugated agarose resin or un-conjugated agarose resin as negative control. As can be seen in Fig. 2, little or no LsHSCARB was detected in the hemin-agarose flow through, whereas an evident band was visible in the negative control flow through. Upon washing of the resin, any protein bound to the agarose was eluted by heat denaturation and sampled for detection by western blot (elution). The most evident band on the membrane is at 100 kDa, which corresponds with the predicted molecular weight of the recombinant protein (100.6 kDa), but there are also fainter bands seen at approximately 65 and 50 kDa. While LsHSCARB clearly could be detected in the hemin-agarose elution, no band was visible in the negative control (Fig. 2), strongly indicating that LsHSCARB is a heme-binding protein. Full length image of the western blot with the protein standard can be seen in Supplementary Fig. S3.

### *In situ* hybridization

**Figure 3:**
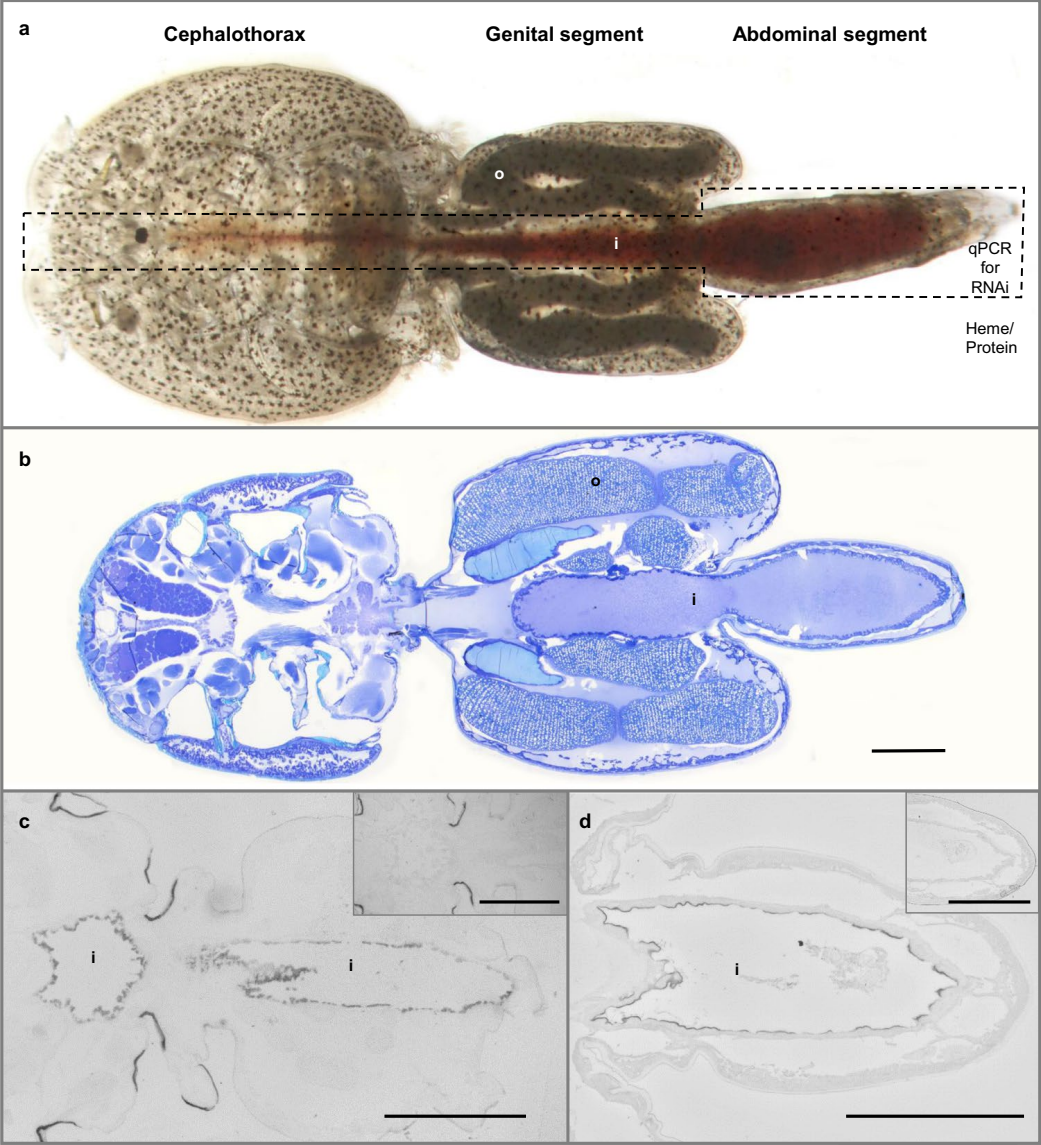
Anatomy of a salmon louse. Photograph of an untreated adult female louse. The blood-filled intestine in the boxed area was dissected out after RNAi to assess knockdown success by qPCR, and the rest of the body was used for heme/protein analyses (**a**). Section of an untreated adult female louse toluidine blue dyed is shown with various structures named. Scale bar: 1 mm (**b**). *In situ* hybridization shows that the transcript of *LsHSCARB* is localized in cells lining the intestinal tract of the adult female salmon louse. The negative sense control in the upper right corner showed no signal. Scale bar: 1 mm (**c**). Immunohistochemical detection of LsHSCARB using anti-LsHSCARB antibody in an adult female louse. Protein is located in the intestine, and the negative control in the upper right corner showed no signal. Scale bar: 500 µm (**d**). Abbreviations: o = oocyte, i = intestine.

To initially characterize the gene, we determined the localization of its transcript by *in situ* hybridization. In the adult female louse, the transcript of *LsHSCARB* was solely detected in cells lining the intestine (Fig. 3c).

### Immunohistochemistry

Immunohistochemistry was used to detect the localization of the LsHSCARB protein in the adult female louse by polyclonal anti-LsHSCARB antibody. The protein was detected in the intestinal border facing the intestinal lumen identical to the location of *LsHSCARB* mRNA (Fig. 3d).

### RNAi mediated ablation of *LsHSCARB*

In order to assess the effect of RNA interference-mediated ablation of *LsHSCARB*, two experiments were implemented. The first was terminated upon extrusion of the second pair of egg string (38 days). At this point, no visible phenotypic alteration was observed. Knockdown efficacies, as well as heme and protein measurements are documented in Supplementary Figs S4–S7. Knockdown efficacy was on average 99% and the heme concentration in knockdown lice was significantly (p = 0.02) reduced by on average 58% compared to control lice. As no visible phenotype alteration was observed despite lower levels of heme, the second experiment was prolonged to observe the effect of an extended period of ablation. Observed values for effect size and standard error from experiment 1 were used as input to conduct power analysis; thus, a minimum n = 8 samples per group was required to achieve adequate power (1-β > 0.80) to detect the observed change in heme concentration, while n = 10 allows for a vast increase in statistical power (1-β > 0.90). The following results are from experiment two.

**Figure 4:**
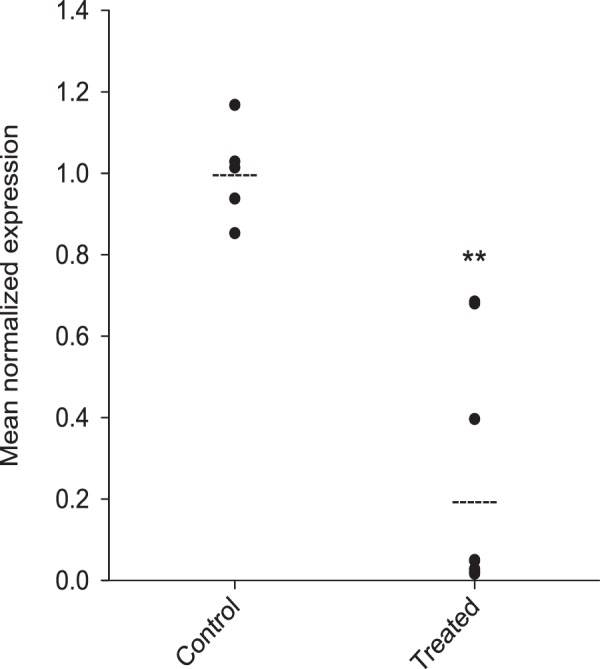
Gene expression analysis by qPCR reveals that *LsHSCARB* is down-regulated 69 days after induced RNAi. The stapled lines indicate the mean value of each group, and the dots represents expression values in individual lice. Asterisks indicate a significant difference between control and treated group. **: significant at p ≤ 0.01. *n* = 5 (control) and 10 (treated).

Knockdown efficacy after injection of double-stranded RNA was assessed in the dissected intestine of each louse (Fig. 3a) by qPCR. At the time point after the extrusion of the fifth egg string pair (observed in the control lice), *LsHSCARB* mRNA was significantly reduced in intestinal tissues of the treated group (Fig. 4). Seven out of ten animals in the treated group had between 95 and 99% reduction in mRNA; the other three animals in the treated group had a reduction of 60, 32 and 32%. All animals were included in the statistical analyses (n = 5 (control) and 10 (treated), p = 0.005).

### Histology and phenotype

**Figure 5:**
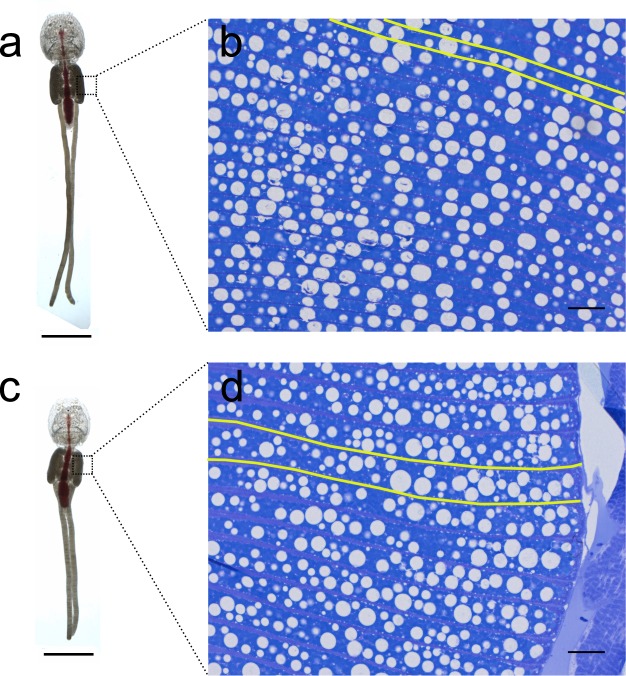
Photographs of representative adult female lice from control (**a**) and *LsHSCARB* knockdown group (**c**). Toluidine blue colored histological sections of oocytes found in indicated areas in genital segments of these lice are shown in right panel for control (**b**) and knockdown (**d**) lice. Yellow lines are drawn along two chorions in each group (**b**,**d**) to indicate the boundaries of one oocyte. Lipid droplets are observed throughout the oocytes. Scale bars: (**a**,**c** = 5 mm, **b**,**d** = 50 µm).

Upon termination of the second experiment, RNAi knockdown animals appeared anatomically normal by visual inspection, although they had shorter egg strings than control animals (Fig. 5a,c). Histological sections of control and dsRNA-treated lice revealed no morphological change other than in the oocytes (Fig. 5b,d). Oocytes from the *LsHSCARB* dsRNA-treated group appear larger and chorions more swollen than in the control group, even though lice from both groups were at the same stage of oogenesis (i.e. egg strings hatched on the same day after terminating the experiment). An annotated histological section of an untreated adult female louse colored with toluidine blue is depicted in Fig. 3b.

### Egg string lengths and offspring development

**Figure 6:**
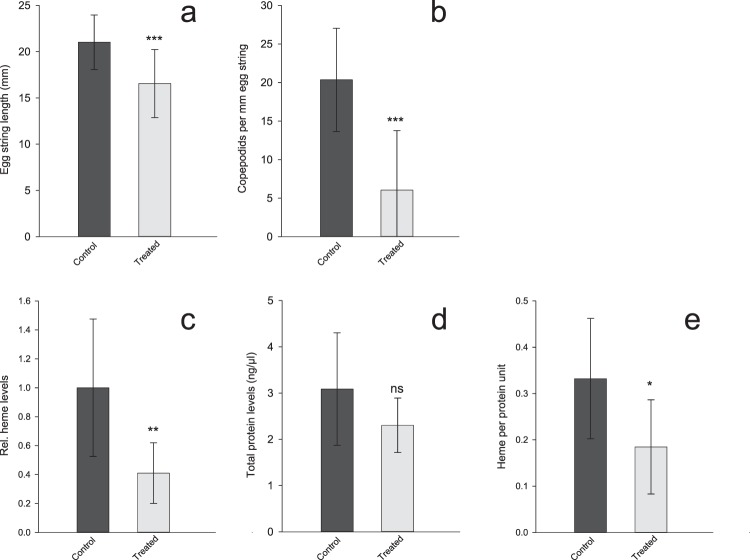
Effect of *LsHSCARB* knockdown. Top: mean egg string lengths (±SD) (**a**) and mean number of copepodids per mm egg string (±SD) (**b**) in control (dark grey) and *LsHSCARB* dsRNA treated (light grey) group (*n* = 19 for control and 12 for treated). Bottom: mean relative heme levels ( ± SD) (**c**). Mean total protein levels ( ± SD) (**d**) and adjusted heme levels to the amount of protein (±SD) (**e**) of lice from the control group (dark grey bars) compared to the *LsHSCARB* knockdown group (light grey bars) (*n* = 10). Asterisks indicate a significant difference between control and treated group. Ns: not significant, *: significant at p ≤ 0.05, **: p ≤ 0.01, ***: p ≤ 0.001.

67% of the control lice were recovered upon termination of the second *LsHSCARB* RNAi experiment. Here, 19 out of 20 lice carried egg strings, with a mean length of 21 mm (Fig. 6a). 50% of the treated lice were recovered at the end of the experiment, and of these, 12 out of 15 lice presented with egg strings with a mean length of 16.5 mm whereas 3 had none (Fig. 6a) (n = 19 (control) and 12 (treated), p = 0.001). Hatching and molting success of the egg strings were monitored. Emerging copepodids were counted, and the number of live animals in relation to their respective egg string length is shown in Fig. 6b (n = 19 (control) and 12 (treated), p = 0.0002). One egg string pair from the control group did not hatch, and three pairs of egg strings did not hatch in the treated group. These are denoted at 0 copepodids per mm egg string, and are included in the calculations (Fig. 6b).

### Heme and protein levels

To measure the effect of RNA interference-mediated ablation of *LsHSCARB* on heme absorption, total heme levels were analyzed from body tissues excluding the intestine that is filled with salmon blood (Fig. 3a). Two animals in the *LsHSCARB* dsRNA-treated group displayed body heme levels below the LOD, and their heme levels were set to the threshold value. Heme levels are significantly lower in the treated animals (Fig. 6c). Total protein levels from the same lysates were slightly, but not significantly (p = 0.089) lower (Fig. 6d). In addition, the adjusted heme levels per protein unit were significantly lower (n = 10, p = 0.011) in the knockdown group compared to the control group (Fig. 6e).

### Starvation

**Figure 7:**
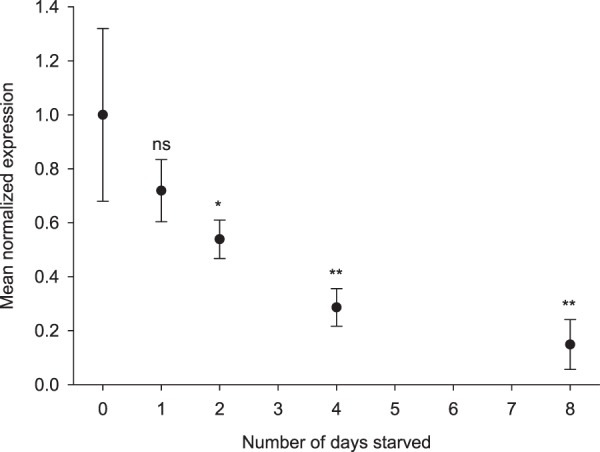
Relative expression of *LsHSCARB* in relation to starvation of adult female lice for 0, 1, 2, 4 and 8 days. Lice taken at 0 days of starvation were used as a calibrator. Asterisks indicate significant difference from day 0. ns: not significant, *: significant at p ≤ 0.05, **: ≤0.01. *n* = 5 per sampling day.

To investigate whether the mRNA expression of *LsHSCARB* in the adult female salmon louse is affected by the presence of host blood in the louse intestine, adult female lice were collected from fish and thereby separated from their food source and starved for zero (sampled immediately), one, two, four and eight days. The expression profile shows a down-regulation in mRNA levels of *LsHSCARB* (Fig. 7) using lice fixed immediately after sampling as calibrator. Down-regulation is significant from day two (day 1: p = 0.124, day 2: p = 0.03, day 4: p = 0.007, day 8: p = 0.003). Compared to lice sampled immediately after being removed from fish, lice starved for eight days have on average an 85% down-regulation of transcript levels of *LsHSCARB*.

## Discussion

As a hematophagous parasite without the option of synthesizing heme on its own, the salmon louse is dependent on acquiring heme from the blood of its host to sustain essential cellular processes. In the present study, we propose a likely mechanism by which the salmon louse takes up heme from its blood-filled intestine. Although the hypothesis about the existence of an intestinal heme receptor has been framed already in 1979^23^, few studies have since identified proteins involved in heme trafficking over intestinal membranes, one of the hindrances potentially being that many animal models have endogenous heme. Using the natural heme auxotrophic parasite *L. salmonis* as a model organism provides a great opportunity to study heme trafficking.

In this study, we have characterized a gene encoding a scavenger receptor class B-like protein that may facilitate heme absorption over the intestinal membrane of the salmon louse. While CD36 is abundant in epithelial cells of the mammalian small intestine and might play a role in gut homeostasis^23^, to the best of our knowledge, homologous proteins have not been previously implicated in heme internalization. Nevertheless, CD36-like receptors bind a multitude of ligands, among them thrombospondin-1, oxidized low-density lipoprotein, beta carotene, long-chain fatty acids, and pathogens^20–22,24^. Other scavenger receptors comprise an even wider family of integral membrane proteins with variety of domain architectures and a broad range of ligands, among them hemoproteins. As an example, Scavenger receptor cysteine-rich type 1 protein M130 (CD163) is involved in removal of the hemoglobin/haptoglobin complex by macrophages^25,26^. Compared to LsHSCARB, CD163 has only a single transmembrane domain and low overall sequence similarity to the receptor characterized here. Membrane topology prediction on the amino-acid sequence of LsHSCARB convincingly yields two transmembrane helices (residues 7 → 28 and 528 → 549), a feature distinguishing class B from all other characterized scavenger receptors. Hence, by domain architecture and by sequence and structural similarity we sustain the hypothesis that LsHSCARB is homologous to class B scavenger receptors.

The ability of recombinant LsHSCARB to bind heme was investigated by a hemin-agarose binding assay, as has also been used for other heme-binding proteins^27,28^. Three bands appear upon detection with anti-LsHSCARB, at approximately 100, 65 and 50 kDa. The band at 100 kDa is the strongest, and corresponds with the predicted molecular weight of the recombinant LsHSCARB with an MBP fusion tag (100.6 kDa). The other two bands are less evident, and could be degradation products, or alternative isoforms of the protein. Our results clearly show that recombinant LsHSCARB is able to bind hemin-agarose resin *in vitro*, but not agarose resin without conjugated hemin, further supporting our hypothesis that it is a heme-binding protein.

3D protein model and the result of *in silico* docking with protoporphyrin IX were also in accordance with our initial hypothesis. We tested the ability of AutoDock Vina to predict binding sites of a variety of known heme-binding proteins for calibration. For the majority of cases, the best binding pose was predicted with high accuracy with respect to RMSD and distance between centroids, giving further confidence that heme ligands could fit into the proposed docking pose geometrically. Of note, for two very similar experimental structures of a homologous receptor to LsHSCARB, the lysosomal integral membrane protein (LIMP), which has not been described as having heme affinity, docking yielded much worse but near identical scores, giving us further confidence in the robustness of the approach. It is further worth noticing that our *in silico* model, unlike crystal structures, does not contain any side-chain information to guide the docking process. While we did not find a known heme-binding motif (e.g. cysteine-proline residues or CXXCH motif), the predicted binding pocket is enriched for non-polar residues (mostly isoleucine and leucine) and contains cysteine-tyrosine (CY) residues on the opposite interior surface, potentially forming the axial ligand to the iron atom of heme. In a comprehensive structural analysis of 125 non-redundant heme-binding protein chains, Li *et al*. (2011) reported that heme-binding pockets were enriched with non-polar residues that create a hydrophobic environment for heme binding, with leucine, isoleucine, and valine slightly more abundant^29^. Besides, the same study also reported enrichment of aromatic residues, whereas we did not observe aromatic residues within the predicted pocket except for the tyrosine residue. Of note, the extracellular domain is strongly enriched for three out of the five common axial ligands of heme compared to LIMP^29^. The structural characteristics of a heme receptor could however be evolutionarily adapted to support a rather transient binding mode compared to other heme-proteins, and this might explain some of the differences observed. Transient binding would further explain slightly lower Vina scores for our receptor, in contrast to proteins with catalytic heme groups, such as nitric oxide synthase, cytochrome P450’s or peroxidases. We further note the presence of multiple residues on the protein surface that have been identified as potentially heme-binding either by prediction or as commonly known axial ligands (Fig. 1b)^29,30^. The existence of multiple unmapped binding modes could contribute to increased efficiency of heme absorptions by binding multiple ligands. More in-depth structural analyses of the receptor in complex with heme are nevertheless required to precisely determine binding modes.

Both transcript and protein of the putative heme scavenger were exclusively expressed in epithelial cells lining the midgut of the salmon louse, further supporting our hypothesis that this protein is important for nutrient absorption from the blood-filled intestine of the salmon louse. Because of the localization of the transcript, we dissected out the intestine to assess knockdown success by qPCR (Fig. 3a). Consequently, the rest of the body could be used for heme and protein measurements. This way, fish-blood in the intestine is excluded and only absorbed heme is measured. As fish blood is highly enriched in heme as seen by the red pigmentation due to heme, measuring heme levels in the blood-filled intestine would otherwise lead to a wrongful estimate of absorbed heme levels.

In the first RNA-interference knockdown experiment (terminated after 38 days) there was no visible phenotypic alteration in the knockdown animals. However, the heme levels of the knockdown animals are significantly lowered. This drove us to prolong the second experiment to investigate how a longer period in the absence of *LsHSCARB* would affect the parasite. The next RNAi mediated knockdown (terminated after 69 days) of *LsHSCARB* also caused a significant reduction in the amount of heme in the tissues of the louse. Moreover, ablation caused lice to have 21% shorter egg strings, and 70% lower hatching success. The two animals with less effective knockdown and egg strings present (32% down-regulation for both) had a 74% greater hatching success than the animals with 95–99% knockdown. We conclude that detrimental effects of nutrient deprivation accumulate over the prolonged knockdown period and manifest in vastly reduced fecundity.

The necessity of heme for development is widely recognized, and the reduction of viable offspring due to heme depletion has been reported on several occasions. The blood fluke, *Schistosoma mansoni*, requires exogenous heme for the production of eggs as incubation with cyclosporin A inhibited heme uptake, and thus reduced fecundity^31^. The requirement of heme for growth is also reported for the hemoparasite *Leishmania tarentolae*^32^. Perner *et al*. (2016) found that serum fed ticks (*Ixodes ricinus*) did not have embryonal development in eggs, whereas adding 10% hemoglobin to the serum rescued embryogenesis^33^. Furthermore, the silencing of a maternal heme-binding protein in the blood sucking insect *Rhodnius proxilus* impaired embryogenesis^34^. The heme measurements in this study indicate that the knockdown animals contain on average 60% less heme than the control animals. The remaining heme could be due to redundant uptake systems within the intestine that should be further investigated. However, since RNA-interference is not able to mediate a complete knockout, remaining transcripts of *LsHSCARB* could lead to residual production of LsHSCARB protein in addition to remaining LsHSCARB protein translated before dsRNA injection. These effects could contribute to continuous, albeit reduced, heme uptake. A passive diffusion of heme across the intestinal border membrane seems unlikely, as free heme is lipophilic and could lead to peroxidation of membrane lipids^5^.

Even though scavenger receptors have not been implicated in intestinal heme absorption before, ablation of similar genes has been done in other blood-feeders. A CD36-like scavenger receptor was knocked down by RNAi in the hard tick *Haemaphysalis longicornis*^35^. The experiment showed impact on blood-feeding behavior, egg production and the hatching rate. It is however unknown whether this is related to heme absorption, as the level of heme was not analyzed. Later, the authors presented that the receptor was involved in granulocyte-mediated microbial phagocytosis in ticks as well as mediating systemic RNAi in ticks^36,37^. We suggest investigating the role of this receptor in the absorption of heme in the mid-gut of the tick further.

Although the ablation of *LsHSCARB* led to shorter egg strings and lower hatching success, histological analyses did not reveal any drastic morphological alteration that could explain these observations. The only difference observed was in the oocytes. In treated animals, the oocytes appear larger and swollen, and also less structured than in the controls, despite the fact that all egg strings hatched on the same day, indicating that oocytes were at a similar stage of maturation. These observations lead to the conclusion that *LsHSCARB* is essential for normal oocyte development in *L. salmonis*.

We further observe that starving animals causes a decrease in the transcript level of *LsHSCARB*. This suggests a positive regulatory feedback system where a nutrient-rich blood-filled intestine leads to induction of gene expression. Down-regulation due to starvation is in contrast to reports from the free-living heme-auxotroph *C. elegans*, where the expression of HRG-1 is up-regulated as a response to decreasing levels of heme^16^. However, the lifestyle of the hematophagous parasite *L. salmonis* differs strongly from that of *C. elegans*. The salmon louse is, unlike *C. elegans*, either on or off its host. Thus, it is either able to feed or not, and will thus not experience a true gradient in heme concentration. Expression of a gene that is solely required for nutrient uptake is energy consuming and thus potentially wasteful under nutrient deprivation. This argument could contribute to explain increased down-regulation under prolonged starvation. In accordance with our findings, a nutrient induced positive feedback mechanism was observed in the crustacean *Macrobrachium nipponense*, where a scavenger receptor was up-regulated by various dietary lipid sources^38^. Further, Staron *et al*. (2017) reported induction of several heme associated proteins under dietary hemoglobin rescue^39^.

In conclusion, the results from functional studies, both *in vitro* and *in vivo,* as well as *in silico* data indicate that *LsHSCARB* is likely encoding a receptor of heme in the salmon louse intestine. The investigation of the CD36-like protein in relation to heme transport shown in this study may further elucidate the trafficking of heme in other species, which as of today is a process that remains enigmatic. Because ablation of the receptor mRNA gives rise to significant reduction of the fecundity of the parasite, the receptor could find its application as a new drug target in pest control of blood feeding parasites. More generally, we have demonstrated the possibility that class B scavenger receptors, among a wide range of other known ligands, could also mediate heme absorption. It remains an open question if this ability is a singular evolutionary event, or if some of the many orthologous sequences in other animals have a similar function. Either way, given that heme is the most bioavailable iron source for humans, medical applications could arise in the future, prospectively for disorders related to iron uptake or the heme biosynthesis pathway.

## Material and Methods

### Sequence analysis, in silico modelling and docking

Initial data for searching transcript and protein sequences and primer design were extracted from the *L. salmonis* genome annotation in Ensembl Metazoa (https://metazoa.ensembl.org/Lepeophtheirus_salmonis). Gene expression data were retrieved from LiceBase (https://licebase.org). Pathway reconstruction was performed using the KEGG Automatic Annotation Server on all predicted protein sequences^40^. All following analyses are based on the GenBank sequence CDW29028.1. Glycosylation prediction was performed using the NetNGlyc 1.0 Server (http://www.cbs.dtu.dk/services/NetNGlyc/) and the NetOGlyc 4.0 Server^41^. Protein BLAST searches were conducted against the GenBank and SwissProt databases^42^. Conserved domain search was conducted using InterProScan^43^ and consensus membrane topology prediction was performed using CCTOP^44^. Prediction of protein 3D-structure was done in i-TASSER^45^, and docking of the best scoring i-TASSER model (model I) with protoporphyrin IX (PPOIX, ZINC26671872) from the ZINC database^46^ was performed using AutoDock Vina with default parameters^47^, using model I as receptor and PPOIX as ligand. Docking was evaluated using experimental heme-containing protein structures and other proteins not known for heme affinity (Supplementary Table S1) after manually removing the heme moiety and non-standard residues, if present. Visualization of structures and docking was done in UCSF Chimera^48^. RMSD and distances between centroids were calculated for all C, N, and O atoms shared between HEM residues in PDB structures and docking poses using UCSF Chimera and the R-package Rpdb (https://cran.r-project.org/package=Rpdb). Enriched residues that could serve as axial ligands to heme (C, M, Y, K, H)^29^ were evaluated in a conserved region only (Supplementary Fig. S1), after pruning potential N- and C-terminal His-tags and terminal gap regions: conserved region was extracted from structural alignment of model I, and PDB ids 4F7B and 4Q4B after re-aligning by MUSCLE^49^. The HemeBIND web-server was used to predict heme-binding residues in model I^50^.

### Plasmid construction and recombinant LsHSCARB protein expression

ORFs coding for the extracellular part of LsHSCARB (residues 31–523), synthesized and subcloned into a pETM41/His-MBP plasmid by GenScript, were transformed into *E. coli* Bl21 De3 cells for protein expression. Cells were grown at 28 °C until reaching an OD600 of 0.6, and protein expression was induced by adding IPTG to a final concentration of 0.5 mM. Proteins were expressed o/n at 18 °C and harvested by centrifugation. Cell pellets were lysed in a buffer (50 mM Tris-HCl pH 7.4, 150 mM NaCl, cOmplete EDTA-free protease inhibitor (Roche), 1 mg/ml lysozyme and either 0.1% FOS16 or 2% DDM), incubated on ice for 30 min and sonicated for 1.5 min with 1 second impulses at 50 amp. The cell sonicate was centrifuged at 15,000 g for 30 min at 4 °C and the supernatant collected. The protein concentration of the supernatant was determined by Direct Detect spectrometer and the supernatant was further used in downstream experiments.

### LsHSCARB binding to hemin-agarose

100 µl of hemin-agarose beads (Sigma, H6390) were washed four times with 50 mM Tris-HCl pH 7.4, 150 mM NaCl prior to use. The beads were then resuspended in 100 µl buffer and added to an Eppendorf tube with 350 µl of the supernatant containing 2.5 µg/µl protein. Incubation was performed on a rotating wheel for one hour at room temperature. After incubation, the flow through was sampled and mixed with SDS-PAGE sample buffer to assess the unbound fraction. The beads were washed four times with wash buffer and finally the beads were incubated in denaturing buffer containing 2% (wt/vol) SDS, 1% (v/v) β-mercaptoethanol, and 500 mM Tris-HCl pH 6.6 for two min at room temperature followed by five min at 99 °C. The beads were centrifuged, and the supernatant was collected for analysis by western blot. Agarose beads with a glutathione ligand (sepharose 4b) were used as negative control and treated in the same manner as the hemin-agarose. Samples were separated on a SDS-PAGE and detected by anti-LsHSCARB on western blot. Initially, the protein was expressed with an N-terminal His-tag. In order to verify that the observed binding to hemin-agarose was not due to His-tag affinity for iron, the His-tag was removed by Q5 site-directed mutagenesis (NEB). The protein without His-tag was used in one of three binding experiments.

### Animals

A laboratory strain of *L. salmonis* was raised on Atlantic salmon (*Salmo salar*) in tanks with seawater (salinity 34.5‰ and temperature 10 °C) as earlier described^51^. Fish were daily handfed a commercial diet and maintained according to Norwegian animal welfare regulations. Experiments were approved by the governmental Norwegian Animal Research Authority (ID8589). Fish carrying lice from RNA interference (RNAi) experiments were kept in single-fish tanks^52^. Egg string pairs of salmon lice were incubated and hatched in single wells in a flow through system^51^ until being used for experiments.

### RNA isolation, cDNA synthesis, and qRT-PCR

All tissues destined for RNA extraction were stored in RNAlater (Ambion) first at 4 °C overnight, and then at −20 °C until usage. Total RNA was isolated from adult female louse intestines or whole animals using TRI Reagent (Sigma-Aldrich) following the manufacturer’s instructions. Samples were homogenized using 5 mm stainless steel beads and a TissueLyser II (Qiagen) for 1 min at maximum frequency. Genomic DNA was digested from the samples by DNase treatment using DNase I (Invitrogen). RNA was quantified and its purity checked by NanoDrop ND-1000 UV-Vis Spectrophotometer (NanoDrop Technologies). Isolated RNA was stored at −80 °C until further use. RNA (300 ng) was reverse transcribed to complementary DNA (cDNA) using the AffinityScript QPCR cDNA Synthesis Kit (Stratagene), and diluted tenfold with RNAse-free water before being stored at −20 °C. *LsHSCARB* knockdown efficiency and the effect of starvation were validated by assessing the transcript levels by quantitative PCR (qPCR) using the previously validated reference gene *LsEF1α*^53^. qPCR was run on a QuantStudio 3 qPCR machine using PowerUp SYBR Green Master Mix (Applied Biosystems) on duplicate samples under standard conditions (50 °C for 2 min, 95 °C for 2 min, 40 cycles of 95 °C for 15 s and 60 °C for 1 min, followed by a melt curve analysis at 60–95 °C). Duplicate samples had a difference in Ct-values < 0.35. A six-point standard curve of twofold dilutions was prepared to assess the PCR efficiency of the assay: E% = (10^1/slope^ – 1) × 100^54^, which was 94% for *LseEF1α* and 100% for *LsHSCARB*. Relative differences (ΔΔCt) in threshold were calculated and transformed by the formula 2^−ΔΔCt ^^55^.

### *LsHSCARB* knockdown by RNA interference

Double stranded (ds) RNA fragments for *LsHSCARB* were generated using the Megascript^®^ RNAi kit (Ambion) with cDNA from an adult female louse as template. A fragment for trypsin (CPY185) from Atlantic cod (*Gadus morhua*) was used as negative control as this has no significant sequence similarity to transcripts of the salmon louse^56^. Primers used for fragment synthesis are listed in Table 1. Fragments were diluted to 600 ng/µl and bromophenol blue was added to visualize successful injections^56^. Knockdown was investigated in female lice only. On the startup day of the experiment, preadult II female lice were removed from reservoir fish by forceps and dsRNA solutions were injected using borosilicate glass capillaries and pressure from a mouth tube. Lice were placed in seawater to recover for four hours before being put on fish held in individual tanks. Ten females and seven males were placed on each fish. For each fragment, three fish in individual tanks were used. The first experiment was terminated after the extrusion of the second egg string pair of mature adult female lice 38 days after injection. The second experiment was terminated upon the extrusion of the fifth egg string pair of mature adult female lice at 69 days after injection. Upon termination, all lice were removed from the fish and photographed under a binocular. Two control lice and three treated female lice were stored in Karnovsky’s fixative, whereas the remaining lice were dissected as shown in Fig. 3a and stored in RNAlater (intestine) and homogenized in lysis buffer (rest of the body). Egg strings, if present on the adult female lice, were incubated in seawater in flow through hatching wells. Egg string hatching time point and the development of the larvae were monitored.

### *In situ* hybridization

The localization of *LsHSCARB* mRNA was detected in the adult female salmon louse by using *in situ* hybridization (ISH) as earlier described^57,58^. An RNA antisense (AS) probe of 731 bp was made from a target specific cDNA template (see Table 1 for primer sequences). A sense (S) probe acted as negative control for the transcript localization, whereas hybridization with a known set of probes detecting *LsTryp1* acted as positive control^59^. The labelled probes were visualized by using anti-digoxigenin (DIG) alkaline phosphatase fragment antigen binding (FAB) fragment (Roche) and a chromogen substrate containing levamisole (Sigma), nitroblue tetrazolium (NTB; Roche) and 5-bromo-4-chloro-3-indolyl phosphate (BCIP; Roche). Microscopy images were captured by an Axio Scope A1 light microscope connected to Axiocam 105 (Zeiss).

### Heme and protein extraction

Whole body tissues of adult female lice excluding intestine (Fig. 3a) were homogenized in 700 µl lysis buffer (50 mM Tris-HCl pH 7.4, 100 mM NaCl, 1% v/v Triton-X-100 (Sigma-Aldrich), 1 × cOmplete™ EDTA-free Protease Inhibitor Cocktail (Roche)) using a mortar and pestle, followed by using a syringe and needle. Homogenates were centrifuged at 12,000 rpm at 4 °C for 15 min. Supernatants were either used directly or stored at −80 °C until use.

### Fluorescent heme quantification assay

The heme quantification method by Morrison (1965)^60^ was adapted to *L. salmonis* and performed as following. Supernatants from heme/protein extractions were diluted 1:20 in 150 µl of 2 M oxalic acid containing 1% w/v iron oxalate (both from Sigma-Aldrich). The solution was divided into two aliquots, one boiled at 99 °C for 30 min and one left at room temperature. By boiling, non-fluorescing heme is reduced to fluorescent protoporphyrin IX^60–62^. The aliquot kept at room temperature was used to control that the tissues did not contain porphyrins naturally. Four parallels of lysis buffer diluted 1:20 and boiled in the oxalic acid solution were used as the blank and to calculate the limit of detection (LOD = 3× standard deviation (SD) of the blank). A positive control of 400 ng/ml hemin (Sigma-Aldrich) (dissolved in DMSO and diluted 1:20 in 2 M oxalic acid containing 1% w/v iron oxalate) was boiled for 30 min and included in three parallels to ensure the samples being within the linear range of the assay (serial dilution from 0–400 ng/ml, Supplementary Fig. S8). A high precision cell made of Quartz SUPRASIL^®^ (Hellma Analytics) held the samples of about 50 µl as they were excited at 406 nm, and emission was read between 600–605 nm using a LS-50B fluorescent spectrometer (Perkin-Elmer). The reading speed was set to 50 nm/min. The cell was rinsed thoroughly with Milli-Q H_2_O between each sample. All samples were read at 25 °C.

### Protein quantification

Total protein levels were quantified using a bicinchoninic acid (BCA) assay with a Bovine serum albumin (BSA) standard (both from Sigma-Aldrich). Two dilutions of each lysate (1:10 and 1:20) were prepared and further diluted in BCA working reagent (1:20) and incubated at 37 °C in a PCR machine for 30 min. Two microliters of the sample were mounted to the instrument and absorbance was read at 562 nm using NanoDrop-1000 spectrophotometer. Negative samples were measured frequently to ensure no protein residues interfered with the experimental sample readings. Protein amounts were used to calibrate heme levels.

### Histology

Animals destined for histological analyses were fixed in Karnovsky’s fixative for a minimum of 24 h at 4 °C. Fixed animals were then rinsed in 1xPBS and dehydrated in 70% EtOH (15 min), 96% EtOH (2 × 15 min), 1:1 absolute EtOH:infiltration solution (1% w/v Hardener I (benzoyl peroxide) in Technovit 7100 resin (Nerliens Meszansky A.S)) (2 h), and incubated overnight in 100% infiltration solution on a shaker. Plastic embedding was done in 15:1 infiltration solution:Hardener II. Two micrometer thick sections were obtained using a microtome (Leica RM 2165) and placed on microscope slides (VWR International). Sections were stained in filtered toluidine blue for 30 s and rinsed thoroughly in H_2_O to remove background stain. Dry slides were mounted with DPX mounting solution (Sigma-Aldrich) and covered with glass cover slips. Microscopy images were captured as described under the *in situ* hybridization section. Images of whole animals were processed and stitched together using an ImageJ plugin as described by Preibisch *et al*.^63^.

### Starvation experiment

Adult female lice were removed from the fish and placed apart from their food source in individual incubators in running sea water, and left there starving for 0 (sampled immediately), 1, 2, 4 and 8 days. Whole animals were sampled in RNAlater according to Trösse *et al*. (2015)^64^. qPCR was conducted as described above.

### Immunohistochemistry

Polyclonal anti-LsHSCARB antibodies (0.8 mg/ml) were produced in rabbits by the company GenScript. The whole extracellular part of the protein was used for the antibody production (PAMIKSQIYENLDLREGTEGFNAFKEPPAPVYLSYSLFHIKNTNEVIRGEPPVLLEVGPYSYRETMRKENLMEQNSRYLSYGKYTKFEFDETNTHKLKCKNRINTPCSKNDKITIINPVLLTLADKLDGLPKTVKDICFEIINNGNEALGIKAEDLFITEEVDKILYTGFDSKSAAIFDKLDTFLMLLLEVIQESLELDIPIKAKDFENIIKIISPAQLSEGTFAFFKGKNATKLQNYYTIENGRFDKESFMNIVEFNGKNKLPEAWWPNVATSITGQLSSEGGSCHRIYGTDGTQFPPFLFNKKKFPLWMFVGELCRTIYVEFESEVEVEGGITAYRYGVGKRVFSMSNPENFCYCQEFFSCAKQTDNDEWDLSQCLKCKDGVMDVSACYGAPIFMSQPHFLQADKEVQAYVKGLEPNSEKHATYLDIEPNLGTPLRAHKKIQINMVLRKVAGIDLLKKVADFRLIPMFWADEGAELDSEKAEELNNVLFSAITIGNT). Paraffin embedded sections (3.0 µm) of an adult female louse were incubated at 60 °C for 30 min and treated with Histo-Clear II (National Diagnostics) for 2 × 10 min. Sections were then rehydrated in ethanol for 3 min per treatment (2 × 100%, 96%, 80%, 50%) and then rinsed in MilliQ H_2_O. Following this, sections were washed 2 × 2 min with TBST (150 mM NaCl, 50 mM TRIS, 0.05% Tween, pH 7.6). Blocking was done with Superblock TBS (ThermoScientific) for 30 min at room temperature before the sections again were washed with TBST for 2 × 2 min. The primary antibody was diluted 1:8000 in TBST and left on the sections for 1 hour at room temperature. The primary antibody was washed off with TBST (2 × 2 min) before incubating with a 1:100 dilution of the secondary antibody (goat anti-rabbit IgG, Sigma-Aldrich) for 30 min at room temperature, and then washed off with TBST (2 × 2 min). Sections were flushed with processing buffer (100 mM Tris-NaCl, 50 mM MgCl_2_, pH 9.5), and then incubated with processing buffer for 10 min. Staining was done with BCIP/NBT Liquid Substrate System (Sigma-Aldrich) for 3 min until a visible color appeared, upon which the reaction was stopped in MilliQ H_2_O. The negative control was incubated with secondary antibody only. Sections were mounted with ImmunoHistomount (Sigma-Aldrich), and images were captured and processed as described under the *in situ* hybridization section. Two individuals were investigated.

### Statistics

All statistical analyses were conducted using R and IBM SPSS Statistics 23 for Windows. The data sets’ parametric requirements were checked by Shapiro-Wilk’s test of normality. Levene’s test was then used to check for equality of variances. Independent two-sample *t*-tests were conducted to evaluate the difference in means between control and experimental groups. For data sets not fulfilling the parametric requirements a Mann-Whitney U test was performed (knockdown effect and number of copepodids after hatching). Power analysis was performed in R using the observed effect size and standard error in experiment 1, assuming a two-sided alternative hypothesis. A p-value ≤ 0.05 was considered statistically significant. Data are presented as mean values ± standard deviations (SD). Graphs were prepared in SigmaPlot 13.0 and processed in Inkscape.

## Supplementary information

Supplementary information

Supplementary Data S1

Supplementary Data S2

Supplementary Data S3

Supplementary Data S4

Supplementary Table S1
