# Supplementary material for: A scavenger receptor B (CD36)-like protein is a potential mediator of intestinal heme absorption in the hematophagous ectoparasite Lepeophtheirus salmonis
Source: Sci Rep. 2019 Mar 12;9:4218. doi: 10.1038/s41598-019-40590-x (PMC6414551; doi:10.1038/s41598-019-40590-x)
Supplement: Supplementary file 1 — Supplementary information [file 41598_2019_40590_MOESM1_ESM.docx]

**A scavenger receptor B (CD36)-like protein is a potential mediator of intestinal heme absorption in the hematophagous ectoparasite *Lepeophtheirus salmonis***

Erna Irene Heggland^1^, Christiane Eichner^1^, Svein Isungset Støve^2^, Aurora Martinez^2^, Frank Nilsen^1^, Michael Dondrup^3*^

*^1^Department of Biological Sciences & Sea Lice Research Centre (SLRC), University of Bergen, Norway*

*^2^Department of Biomedicine & K.G. Jebsen Centre for Neuropsychiatric Disorders, University of Bergen, Norway*

*^3^Department of Informatics & Sea Lice Research Centre (SLRC), University of Bergen, Norway*

**Corresponding author*

*Dr. Michael Dondrup*

*University of Bergen,*

*Thormøhlensgate 55*

*5008 Bergen*

*Norway*

*+47 555 84 354*

[*Michael.dondrup@uib.no*](mailto:Michael.dondrup@uib.no)

**Supplementary information includes:**

**Supplementary Figures S1-S8, Supplementary Data S1-S4 and Supplementary Table S1**

Supplementary Figures:

Supplementary Figure S1:

Alignment of amino-acid sequence of LIMP-2 (first row) and LsHSCARB (second row).


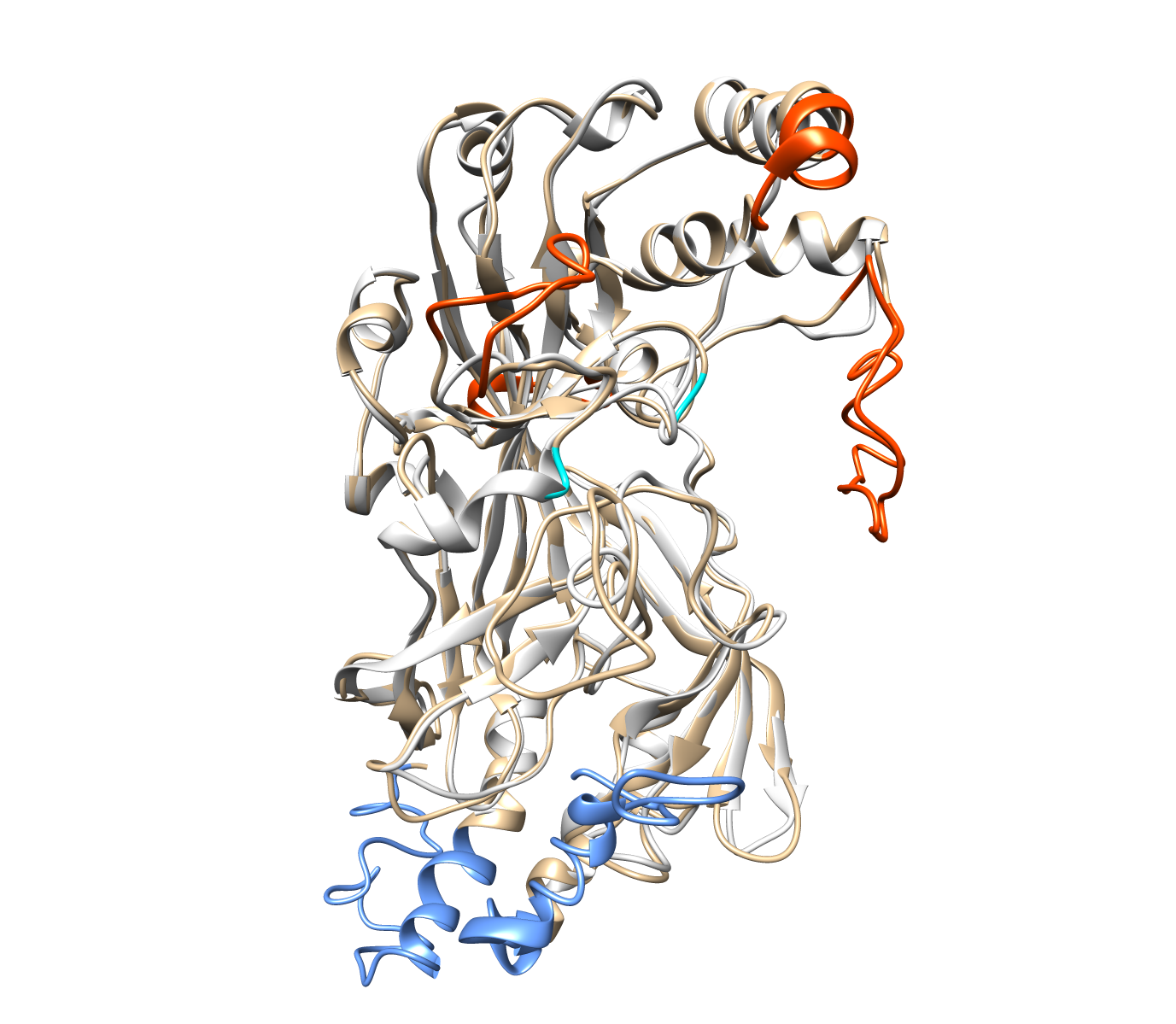


Supplementary Figure S2:

Structural alignment of LsHSCARB model1 (silver) vs the most used template structure (LIMP-2, gold) in ribbon representation. Extracellular residues in insertions of model1 in comparison to LIMP are highlighted in red, insertions in intra-cellular and trans-membrane regions are highlighted in blue. Two CY residues are highlighted in cyan.


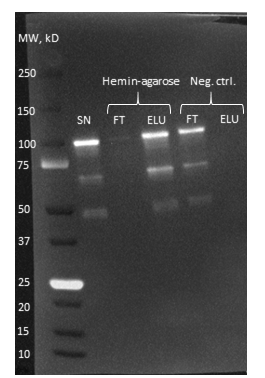


Supplementary Figure S3:

Full length image of western blot from the hemin-agarose pull down assay including precision plus protein dual color standard (Bio-Rad). SN = supernatant, FT = flow through, ELU = elution. Exposure time = 29.5 sec.

Supplementary Figure S4:

Knockdown effect of double stranded *LsHSCARB* measured in RNAi experiment 1 by qPCR. Mean ± SD. n = 5 (p = 0.009)

Supplementary Figure S5:

Heme levels measured by fluorescence in RNAi experiment 1. Mean ± SD. n = 5 (p = 0.02)

Supplementary Figure S6:

Protein levels measured by BCA assay in RNAi experiment 1. Mean ± SD. n = 5 (p = 0.07)

Supplementary Figure S7:

Heme/protein levels in RNAi experiment 1. Mean ± SD. n = 5 (p = 0.045)

Supplementary Figure S8:

Standard curve of 0-400 ng/ml hemin in DMSO. Measured by fluorescence intensity (excitation: 406 nm, emission: average of 600-605 nm) using a LS-50B fluorescent spectrometer (Perkin-Elmer).

Supplementary Data:

Supplementary Data S1: (file: scarb-ppoIX-dock.receptor.pdb)

Input files to Autodock Vina: receptor model corresponding to LsHSCARB (model1) used for docking in PDB format.

Supplementary Data S2: (file: scarb-ppoIX-dock.ligand.pdb)

Input files to Autodock Vina: ligand model corresponding to Protoporphyrin IX (ZINC26671872) used for docking in PDB format.

Supplementary Data S3: scarb-ppoIX-dock.conf

Input files to Autodock Vina: Configuration file used for the docking analysis.

Supplementary Data S4: scarb-ppoIX-dock.pdbqt

Output file from docking of LsHSCARB and Protoporphyrin IX by Autodock Vina in PDBQ format.

Supplementary Table:

Supplementary Table S1: re-docking-resultsPPOIX-1.xlsx

Results from the docking experiments including known heme-binding structures from PDB sorted by best docking score from AutoDock Vina. RMSD and distance of centroids are given between the best docking pose and the ligand location in the experimental structure for the C, N, and O atoms shared between ligands.
